# A Chinese CADASIL family with a rare heterozygous mutation in exon 2 of *NOTCH3*: A case report

**DOI:** 10.1097/MD.0000000000040107

**Published:** 2024-10-11

**Authors:** Jingrong Guo, Lulu Liu, Minli Yan

**Affiliations:** a Department of Neurology, The First Affiliated Hospital of Henan University of Chinese Medicine, Zhengzhou, China; b School of the First Clinical Medicine, Henan University of Chinese Medicine, Zhengzhou, China; c Department of Neurology, The Third People’s Hospital of Luoyang, Luoyang, China; d Department of Neurology, The First Affiliated Hospital of Zhejiang Chinese Medical University (Zhejiang Provincial Hospital of Chinese Medicine), Hangzhou, China.

**Keywords:** CADASIL, exon 2, novel mutation, the *NOTCH3* gene

## Abstract

**Rationale:**

Cerebral autosomal dominant arteriopathy with subcortical infarcts and leukoencephalopathy (CADASIL) is an inherited cerebrovascular disease caused by the neurogenic locus notch homolog protein 3 (*NOTCH3*) gene mutation. In recent years, most of the newly reported mutations of CADASIL patients mainly occur in exon 3 to 24, while the cases related to exon 2 mutation are rare, and clinical research data are relatively insufficient. In this study, we have reported a case of a rare heterozygous mutation c.128G>A (p.Cys43Tyr) in exon 2 of *NOTCH3* in a 41-year-old Chinese man in the light of relevant literatures.

**Patient concerns:**

A 41-year-old man who suffered slurred speech for 5 days and right lower limb weakness for 4 days was admitted to our hospital.

**Diagnoses:**

Magnetic resonance imaging of the head revealed diffuse white matter lesions involving the outer capsular area and bilateral temporal poles. The rare heterozygous mutation c.128G>A (p.Cys43Tyr) in exon 2 of *NOTCH3* was identified through molecular genetic testing. The proband was diagnosed as having CADASIL. Meanwhile, the same mutation was detected in 2 other family members III5 and IV9.

**Interventions:**

Atorvastatin calcium tablet (20 mg qd) and aspirin enteric-coated tablet (100 mg qd).

**Outcomes:**

The patient was hospitalized for 3 weeks and discharged after his symptoms improved.

**Lessons:**

The heterozygous Cys43Tyr mutation in exon 2 of *NOTCH3* is rare. Thus, our case report complements the rare mutation of exon 2 and offers additional clinical data for CADASIL patients.

## 1. Introduction

Cerebral autosomal dominant arteriopathy with subcortical infarcts and leukoencephalopathy (CADASIL) is a rare monogenic inherited disease caused by a mutation in the notch homolog protein 3 *(NOTCH3*) gene on chromosome 19p13.12 region; it is the most common hereditary stroke disease.^[1,2]^ More than 400 *NOTCH3* mutations have been reported globally.^[3]^ Generally, the clinical manifestations of CADASIL are observed in adulthood (age: 40–50 years). These manifestations chiefly include migraine with aura, recurrent ischemic strokes, progressive subcortical dementia, cognitive dysfunction, and mental and emotional disorders. Brain magnetic resonance imaging (MRI) of CADASIL patients presents diffuse leukoencephalopathy, multiple lacunar infarctions, and cerebral micro-bleeds. The genetic test conducted to identify *NOTCH3* mutations remains the sole gold standard for CADASIL diagnosis.

We have reported here the case of a rare missense mutation of *NOTCH3* in a Chinese family with CADASIL and discussed its clinical manifestations, including the results of MRI and gene mutation analysis.

## 2. Case report

A 41-year-old man who suffered slurred speech for 5 days and right lower limb weakness for 4 days was admitted to our hospital. Two months before admission, he developed cerebral infarction. His blood lipid levels were high. He had a more than 10-year history of migraine headaches, but no history of hypertension, diabetes, and coronary heart disease. He did not smoke and never drank alcohol.

**Figure 1.**
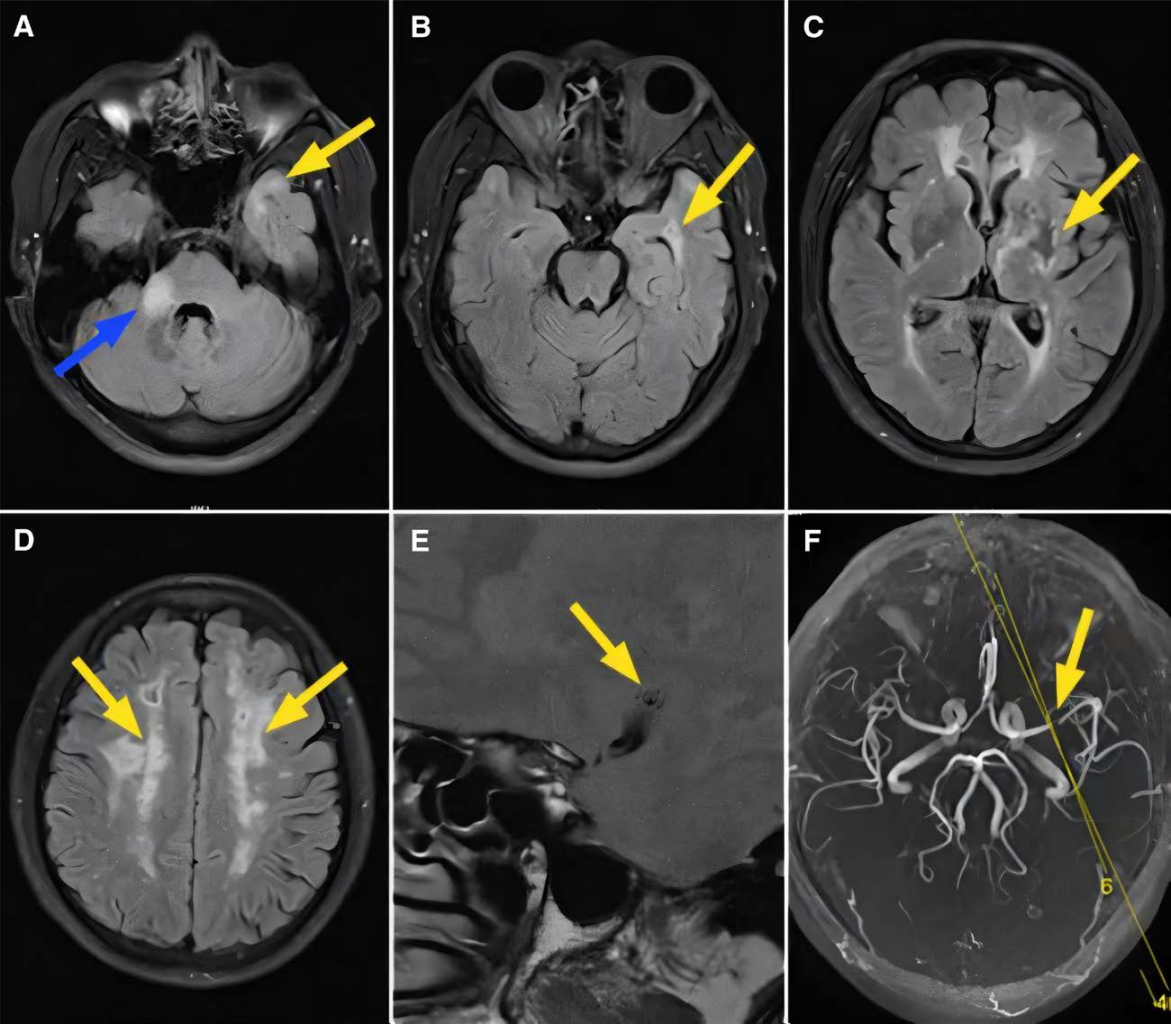
Brain magnetic resonance imaging of the proband. (A) FLAIR image showed acute infarction in the right cerebellar root (Blue arrow), and white matter lesion in the temporal region (yellow arrow). (B and C) FLAIR image showed temporal white matter lesions and external capsule lesions (yellow arrow). (D) FLAIR image showed extensive fused white matter high signal in the bilateral hemianopia (yellow arrow). (E and F) High-resolution MR + MRA of the brain revealed severe stenosis of the left middle cerebral artery, and the inferior plaque of the stenosis was strengthened, yellow line is E positioning line.

**Figure 2.**
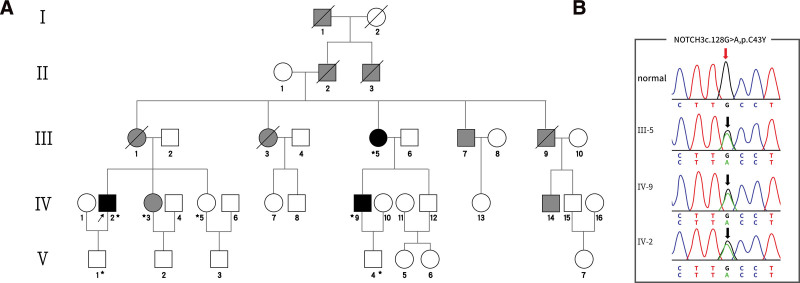
Family lineage and gene sequencing results. (A) Family lineage. Squares represent men and circles represent women. The arrow indicates the proband (IV2). Gray represents people with cerebral infarction or mental disorders. Black indicates affected members. Slash indicates deceased members. Gene-tested subjects are marked with an asterisk. (B) Gene sequencing results. The *NOTCH3* gene sequencing results identified the variant of c.128G>A (p.Cys43Tyr) mutation in exon 2 was detected in the proband (IV2) and 2 other family members III5 and IV9.

Physical examination revealed slurred speech and grade 4 muscle strength in the right lower limb. The results of other neurological examinations were unremarkable. The total blood cholesterol concentration was 6.01 mmol/L (normal range: 3–5.2 mmol/L), and the triglyceride concentration was 2.67 mmol/L (normal range: 0.45–1.69 mmol/L). On brain MRI and fluid attenuation inversion recovery (FLAIR) imaging, multiple ischemic foci with bilateral semioval centers and ventricular voids, and acute foot infarction in the right cerebellum were noted (Fig. 1A–D). Brain magnetic resonance angiography (MRA) presented severe stenosis of the left middle cerebral artery, with plaque enhancement under the stenosis (Fig. 1E–F). Based on the clinical and neuroimaging findings, the patient was clinically suspected of CADASIL. After receiving the patient’s informed consent, the analysis of targeted multi-gene sequencing for cerebrovascular genetic diseases was conducted. Primer sequences were designed using the reference sequence NM_000435.2. Variant results revealed a heterozygous mutation c.128G>A (p.Cys43Tyr) in exon 2 of *NOTCH3* (Fig. 2B). After the patient was admitted to the hospital, he was administered symptomatic treatment such as atorvastatin calcium tablet (20 mg qd) to reduce lipid levels and stabilize plaque and aspirin enteric-coated tablet (100 mg qd) to prevent platelet aggregation, improve brain circulation, and remove oxygen free radicals. The patient was hospitalized for 3 weeks and discharged after his symptoms improved.

Since then, family genealogies have been established through medical and genetic surveys of the proband families (Fig. 2A). The proband’s mother (III) had a history of cerebral infarction, but a genetic analysis could not be conducted as she had committed suicide. Other family members (I1, II2, II3, III3, III5, III7, and III9) also had a history of cerebral infarction or mental disorders (IV3 and IV14). The Family Member IV9 had a history of migraines. After informed consent was obtained, blood was collected from the family members V1, V4, IV3, IV5, IV9, and III5. According to gene sequencing analysis, an identical heterozygous mutation c.128G>A (p.Cys43Tyr) was presented in exon 2 of *NOTCH3* in subjects III5 and IV9 (Fig. 2B). In contrast, other family members tested negative.

## 3. Discussion

CADASIL is a small artery disease predominantly affecting the brain. It is among the most common inherited cerebrovascular diseases in adults. CADASIL presents varied clinical manifestations, with common symptoms including recurrent migraine, subcortical ischemic events, cognitive impairment, and psychiatric symptoms.^[1,4]^ These symptoms can progress to severe vascular dementia. Brain MRI shows diffuse white matter lesions, multiple subcortical lacunar infarction, and cerebral microhemorrhage. A characteristic bilateral white matter involvement is noted in the temporal pole and external capsule. Additionally, MRI shows that brain atrophy is another crucial feature of CADASIL. It may partly be caused by subcortical ischemic lesions damaging connecting fibers, leading to secondary neurodegeneration in the cortical region. Consistently, the brain MRI of the proband presented with typical diffuse white matter lesions involving the external capsule and bilateral temporal poles. MRI presenting a diffuse hyper-signal in the temporal pole is a beneficial diagnostic marker. On investigating 83 CADASIL patients, Markus HS found that the specificity and sensitivity of MRI for moderate or severe anterior temporal pole injury in CADASIL diagnosis were 86% and 89%, respectively.^[5]^ Interestingly, high-resolution MR + MRA of the proband’s head suggested severe stenosis of the left middle cerebral artery. Relevant studies have shown that small artery disease is the main manifestation, but large artery disease may also be 1 of the manifestations of CADASIL.^[6,7]^ In our case, considering that the proband had cerebrovascular risk factors including hyperlipidemia, a part of our observation may be related to concomitant presence of intracranial large vessel atherosclerosis. Therefore, MRI is a crucial tool for the clinical diagnosis of CADASIL. Performing brain MRI in all patients with suspected CADASIL is strongly necessary.

CADASIL is caused by *NOTCH3* mutations on chromosome 19p12.13 with 33 exons. CADASIL-related mutations exist in exons 2 to 24 of *NOTCH3* encoding epidermal growth factor (EGF)-like domains.^[8]^ Abnormal accumulation and deposition of mutated Notch3 protein aggregates on the surface of VSMCs, pericytes, and endothelial cells are the key pathophysiological mechanisms underlying CADASIL. Likely pathogenic variants are mostly located within EGF-like repeat (EGFr) domains in the extracellular part of the Notch3 receptor.^[9]^ About 95% of genetic mutations are caused by missense mutations, while intraframe deletions, frameshift deletions, and splice site mutations are relatively uncommon.^[10]^ EGFr domains possess cysteine residues that are critical for maintaining the protein’s disulfide bonds and structural and functional stability. Missense mutations affecting the cysteine residue number may induce structural and functional abnormalities in the *NOTCH3* protein.^[11]^ If a patient is suspected of CADASIL, genetic testing can be performed, including single gene detection (*NOTCH3* sequence analysis and target gene deletion/duplication analysis). If conditions permit, multi-gene combination detection, including *NOTCH3* and other target genes associated with stroke risk, can be performed.

The initial symptoms, onset age, and disease progression of CADASIL vary between and within families. The onset age and severity of clinical manifestations were positively associated with the mutation type and location in particular exons. It has recently been shown that *NOTCH3* EGFr 1 to 6 pathogenic variants may cause a more severe phenotype with earlier age at stroke onset, lower survival time, and greater brain MRI lesions when compared with EGFr 7 to 34 pathogenic variants.^[12,13]^ However, the genotype-phenotype association has not been firmly established. In a multicenter retrospective study, carriers of the mutations p.Arg607Cys or p.Arg544Cys in exon 11 had a significantly lower frequency of migraine with aura, were more susceptible to cognitive disorders, and had an older onset age.^[14]^ In our case, gene sequencing indicated that 3 Chinese family members had a pathogenic missense mutation of c.128G>A (p.Cys43Tyr) in exon 2 of *NOTCH3*. The proband and multiple family members presented with migraines, recurrent ischemic strokes, or mental disorders. These presentations were highly compatible with the phenotypic spectrum of CADASIL reported in previous studies.^[15,16]^ However, of note, among patients with exon 2 mutations reported in the past, some initial symptoms were vertigo, tinnitus,^[17]^ and acute disturbance of consciousness,^[18]^ whereas some patients were even asymptomatic.^[19]^ Most patients had a family history of stroke or dementia, while further neuroimaging examination demonstrated typical disease features of CADASIL. This fact indicates that many atypical features of CADASIL also exist in clinical practice, which brings great challenges to the clinical diagnosis of this disease. CADASIL can be easily misdiagnosed or missed clinically, thereby resulting in a higher actual incidence than that reported. In our case, 3 confirmed CADASIL patients from the same family carrying the same *NOTCH3* mutation had different clinical phenotypes, but all had a history of hyperlipidemia. The family members IV9 and III5, respectively, had a history of smoking and drinking and hypertension. Other factors, such as environmental factors, lifestyle, diet, medication, and other vascular risk factors, may have had an impact on the severity of disease presentation.^[20,21]^

CADASIL diagnosis is usually combined with a positive family history of migraine/stroke/dementia, typical white matter changes on MRI findings, and *NOTCH3* detection. A diagnosis of CADASIL can be made when molecular gene detection identifies the presence of the heterozygous pathogenic variation of *NOTCH3*. However, genetic testing did not detect all CADASIL patients. Exon sequencing detected no likely pathogenic variants in up to 4% of all patients.^[22]^ Electron microscopy displaying granular osmophilic material deposits on the skin or VSMCs surface is also a diagnostic modality. The specificity of skin biopsy is close to 100%, albeit the sensitivity varies.^[23,24]^ Therefore, if clinical symptoms and imaging results indicate a high suspicion of CADASIL, and the genetic test is negative (or not conditional), a skin biopsy is useful. Currently, no specific treatment is available to cure CADASIL. Management strategies implemented for CADASIL focus on symptomatic relief, prevention of stroke and other vascular events, and supportive care. Lifestyle modifications, including blood pressure control, antiplatelet therapy, and regular monitoring, are also essential for CADASIL management.

This case has some limitations, as several of the proband’s family members were already dead (I1, II2, II3, III1, III3, and III9), and several members suspected of having CADASIL could not be genetically evaluated. The proband’s sister (IV3) had a history of psychiatric disorders, but the genetic test was negative. In fact, further brain MRI or skin biopsy was required to eliminate CADASIL. However, with due consideration to personal wishes of the patient’s family, we could not do so. In addition, the proband and 2 other family members (III5 and IV9) need to be followed up to obtain more meaningful clinical data and offer a valuable reference for CADASIL prognosis and treatment.

## 4. Conclusion

In summary, we have described 3 patients with heterozygous mutations c.128G>A (p.Cys43Tyr) in exon 2 of *NOTCH3* from China. The findings complement the rare mutation of exon 2 and provide additional clinical data for CADASIL patients. CADASIL-related clinical manifestations are complex and varied. Therefore, CADASIL can be easily misdiagnosed or missed. In clinical practice, carefully inquiring about the patient’s family history, comparing various clinical symptoms, and combining neuroimaging characteristics are necessary to further confirm the diagnosis through skin biopsy and genetic testing.

## Acknowledgments

We thank the patient and his family for their cooperation.

## Author contributions

**Data curation:** Jingrong Guo, Lulu Liu.

**Software:** Lulu Liu.

**Supervision:** Minli Yan.

**Writing – original draft:** Jingrong Guo.

**Writing – review & editing:** Jingrong Guo.
